# A Scoping Review of Policies Related to the Prevention and Control of Overweight and Obesity in Africa

**DOI:** 10.3390/nu13114028

**Published:** 2021-11-11

**Authors:** Theodosia Adom, Anniza De Villiers, Thandi Puoane, André Pascal Kengne

**Affiliations:** 1 Nutrition Research Centre, Radiological and Medical Sciences Research Institute, Ghana Atomic Energy Commission, Accra LG80, Ghana; 2 Division of Health Systems and Public Health, Department of Global Health, Faculty of Medicine and Health Sciences, Stellenbosch University, Cape Town 7505, South Africa; 3 Non-Communicable Disease Research Unit, South African Medical Research Council, Cape Town 7505, South Africa; Anniza.DeVilliers@mrc.ac.za (A.D.V.); Andre.Kengne@mrc.ac.za (A.P.K.); 4 School of Public Health, Faculty of Community and Health Sciences, University of Western Cape, Cape Town 7535, South Africa; tpuoane@uwc.ac.za; 5 Department of Medicine, Faculty of Health Sciences, University of Cape Town, Cape Town 7700, South Africa

**Keywords:** policy, programmes, NCDs, overweight/obesity, ANGELO framework, unhealthy diets, physical inactivity, Africa

## Abstract

To address the issue of obesity, the World Health Organization (WHO) recommends a set of comprehensive programmes aimed at changing the obesogenic environments to provide opportunities for healthy food options and increased physical activity in the school, home, and at the population level. The objectives of this study were to examine the nature and range of policies related to overweight and obesity prevention in Africa, and to assess how they align with international guidelines. An existing methodological framework was adapted for this scoping review. A search of publicly available national documents on overweight/obesity, general health, and non-communicable diseases (NCDs) was undertaken from relevant websites, including WHO, ministries, and Google Scholar. Additional requests were sent to key contacts at relevant ministries about existing policy documents. The documents were reviewed, and the policies were categorised, using the Analysis Grid for Environments Linked to Obesity (ANGELO) framework. The framework categorises the environmental drivers of obesity into four domains (physical, economic, legislative, and socio-cultural) and two scales: macro (national, regional, sectors, food industries, media, etc.) and micro (household, institutional, and community). This review included documents from 41 African countries. The policy initiatives to prevent overweight/obesity target the school, family and community settings, and macro environments, and broadly align with global recommendations. The NCD documents were in the majority, with only two on obesity. The majority of the documents detailed strategies and key interventions on unhealthy diets and physical inactivity. The physical, legislative, and sociocultural domains were largely featured, with less emphasis on the economic domain. Additionally, nutrition- and diet-related policies were in the majority. Overlaps and interactions of policies were observed in the application of the ANGELO framework. This study has provided information on national policies and programmes in Africa and can be useful as a first point of call for policymakers. The overlapping and interaction in the initiatives demonstrate the importance of multi-sectoral partnerships in providing supportive environments for healthy behaviours.

## 1. Introduction

Globally, an estimated 18% of children and adolescents aged 5–19 years and 39% of adults were overweight or obese in 2016 [1]. The available statistics show increasing trends of body mass index and obesity in Africa. For example, between 1984 and 2014, the age-standardised mean BMI increased in adult men from 21.0 kg/m^2^ to 23.0 kg/m^2^, and from 21.9 kg/m^2^ to 24.9 kg/m^2^ in women [2]. Similarly, recent estimates from the Global Burden of Disease, Injuries, and Risk Factors Study revealed that obesity more than doubled in Southern African populations, from 12.0% to 18.5% in adult females and 4.5% to 8.8% in adult males between 1990 and 2019. Moreover, among children and adolescents aged 2–19 years, the prevalence rates increased from 2.4% to 5.5% in boys and from 2.8% to 6.0% in girls over the period [3].

The focus has been on unhealthy diets and physical inactivity as the main determinants of the growing obesity crisis [4,5]. However, with the moderate success of individual lifestyle interventions to prevent and control overweight/obesity [6,7,8,9,10], recent attention has been shifted to the wider structural, global and national systems as significant drivers of the obesity crisis [11]. A considerable number of research has linked changes in the food system resulting from global and international food trade to the nutrition transition observed in low-to-middle income countries [12,13,14]. At the same time, structures and national policies on urban planning and design impact neighbourhood walkability, public transport, and public amenities for recreation [11].

To address the issue of obesity, the World Health Organization (WHO) [15,16,17] proposes comprehensive population-wide policy actions and plans for member states, aimed at changing obesogenic environments to provide opportunities for healthy food choices and increased physical activity at the school, home, workplace, healthcare facilities, community, and population levels. It is the responsibility of governments to provide political leadership and commitment by developing multi-sectoral policies and programmes, given that no single intervention is adequate to meet the prevention and control objectives. This scoping review therefore aimed to examine the nature and range of policies related to overweight/obesity prevention in Africa, and to assess how they align with international guidelines for the prevention of obesity.

## 2. Materials and Methods

The methodological framework for scoping review proposed by Arksey [18] was adapted to develop the protocol for this review, the details of which are provided elsewhere [19].

### 2.1. Stage 1: Identifying the Research Questions

Based on the literature, the WHO documents, and in the context of this review, key research questions were derived. These covered investigating policy actions undertaken by African countries to reduce unhealthy diets and physical inactivity to prevent overweight/obesity.

### 2.2. Stage 2: Identification and Collection of Policy Documents

Briefly, a search of relevant, publicly available national documents on overweight/obesity, health, nutrition, and or non-communicable diseases (NCDs) was undertaken from Google Scholar, WHO websites, and relevant ministries in countries in Africa, using the key words ‘nutrition’, ‘food’, ‘physical activity’ in combination with ‘policy’, guideline’, ‘action plan’, ‘programmes’, ‘strategy,’ ‘regulation’, ‘law’, relating to ‘overweight’, ‘obesity’, and ‘non-communicable diseases’. Additionally, requests were sent to key contacts at the health and education ministries of the countries about existing policy documents. The last search date was 29 April 2019 and covered the period of 2003 to 2019.

### 2.3. Stage 3: Screening and Selection of Policy Documents

This was done following pre-determined inclusion and exclusion criteria. Documents were included if they were national policies, either adopted and/or at the draft stage; initiatives were being implemented or actions and strategies were proposed, targeted at unhealthy diets and physical inactivity; they formed part of a larger chronic disease prevention strategy to prevent and control overweight/obesity; and were post 2000. No language restrictions were set. Documents that were not national in coverage and reviews of policies were excluded. Where updated duplicate documents were found, the most recent, in terms of the year of production, was included.

### 2.4. Stage 4: Charting the Data

The documents were reviewed, and a standardised form was used to extract data on the nature of policies, e.g., title of document, type (policies, programmes, strategic plans or strategies, and action plans), year of publication or production and status (whether documents had official signatories, or at the draft stage), and coverage, such as policies to increase fruit and vegetable intake, limit the intake of fat and sugar, and promote physical activity.

### 2.5. Stage 5: Collating, Summarising, and Reporting the Results

For the purposes of this review, policy refers to all documents regardless of the type, and includes policies, programmes, strategic plans or strategies, and action plans. The policies were categorised using the Analysis Grid for Environments Linked to Obesity (ANGELO) framework [20], commonly used for understanding the obesogenic environment. The ANGELO framework was first applied by Swinburn et al. [20] to study the environmental determinants of obesity in some Pacific Island communities with the highest rates of overweight and obesity globally. The framework categorises the environmental drivers of obesity into four domains (physical, economic, legislative, and socio-cultural). The drivers are further divided into two scales: macro (national, regional, sectors, food industries, media, etc.) and micro (households, institutions, and community). While policies at the micro level are context specific, the macro-level policies are necessary to ensure effectiveness and sustainability at the population level.

The physical environment refers to the availability of facilities, built environment, training opportunities, nutrition and exercise expertise, and information. Economic environment relates to monetary considerations associated with diet and physical activity, including taxes and incentives. Legislative environment comprises rules and regulations, guidelines, and policy messages on diet and physical activity. Socio-cultural refers to attitudes, beliefs, culture, and social norms relating to diet and physical activity; Table 1. Based on the contents of these documents, the policy actions on diet and physical activity were manually mapped. Those policy actions that were context specific were assigned to one of the four domains under micro scale. Likewise, all policies that were targeted at the general population were categorised as macro. The results are presented by the settings, key policy interventions, policy domains, scale, and by country.

### 2.6. Ethics Consideration

Ethics approval was not required since the study did not involve the collection of primary data. The results are based on data from publicly available documents.

## 3. Results

### 3.1. Description of Policy Documents

**Figure 1:**
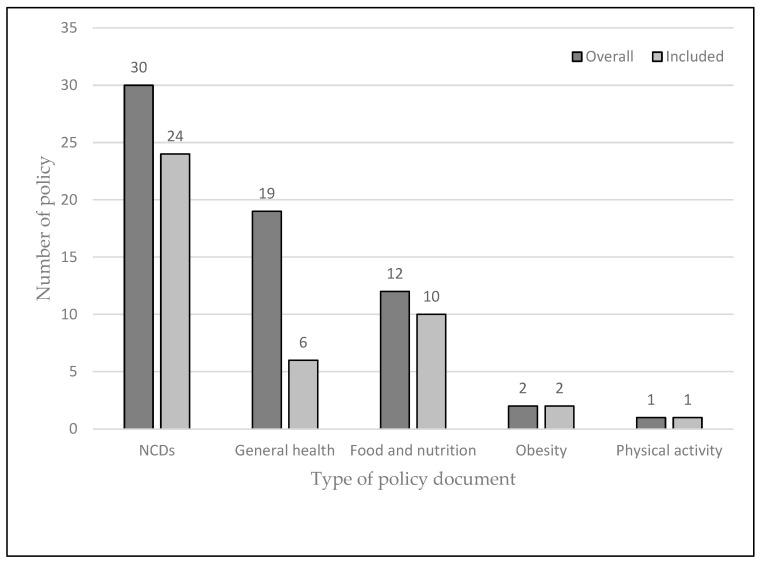
National policies reviewed, overall and included.

The searches resulted in 87 documents from 54 African countries. Out of this, 64 documents were reviewed, and 43 documents from 41 countries were included in this study (Figure 1, Supplementary Table S1). After initial searches, it became apparent that many countries did not have standalone policies on obesity prevention, so the searches were modified to focus on NCDs. Thirty (46.9%) of the retrieved documents were on NCDs [21,22,23,24,25,26,27,28,29,30,31,32,33,34,35,36,37,38,39,40,41,42,43,44,45,46,47,48,49,50], 19 (29.7%) on general health [51,52,53,54,55,56,57,58,59,60,61,62,63,64,65,66,67,68,69], 12 (18.8%) on nutrition [42,70,71,72,73,74,75,76,77,78,79,80], 2 (3.1%) on obesity [81,82], and 1 (1.5%) on physical activity [83]. In addition to the obesity and physical activity policies, 24 on NCDs, 6 on general health, and 10 on food and nutrition were included. Furthermore, these documents were described as policies, strategies, strategic plans, or action plans. Thirteen countries, namely, Burundi, Cape Verde, Comoros Island, Democratic Republic of Congo, Djibouti, Eritrea, Mali, Sao Tome et Principe, Senegal, Somali, South Sudan, Sudan, and Uganda [27,32,38,50,54,55,61,62,63,64,67,69,73], did not detail policy measures to address unhealthy diets and physical inactivity, although the policies underscored the importance of these risk factors. Table 2 summarises the key policy actions identified in the reviewed documents.

### 3.2. Key Policy Interventions at the Micro Scale (School, Family and Community)

These included the provision of healthy school meals, promotion of school vegetable gardens, marketing of unhealthy food and beverages in and around the school, integration of the concepts of nutrition and heathy eating in the curricula, professional development for teachers, canteen staff and school doctors, and monitoring of body mass index (BMI).

The majority (51.2%) of the documents outlined actions to strengthen nutrition education of children. The provision of healthy school meals was addressed in 18 countries. For example, Botswana, Kenya, Nigeria, Guinea-Bissau, and Liberia targeted healthy school meals as a component of the school health programme [22,35,40,74,84]; Algeria and Seychelles specified the free distribution of fruits and drinking water fountains [21,43]; and others, including Benin and Ghana, proposed the mandatory inclusion of fruits and vegetables in the school menu [23,33]. The promotion of vegetable gardens was specified by six countries [21,42,74,75,76,81], while Algeria [21], and South Africa [82] targeted regulations on the sugar, fat, and salt content of food sold near schools. Monitoring the BMI of children [21,22,48], professional development for staff [21,46,74,76], and marketing of unhealthy foods [21,33,70,81,82] were addressed in three, four and five country documents, respectively.

The promotion and strengthening of mandatory physical education and or physical activity in the curriculum as strategies to promote physical activity was mentioned by 19 countries, including Algeria [21], Botswana [22], Chad [26], Ghana [33], Seychelles [43], Nigeria [40], and South Africa [82]. Two countries, Mauritius [83] and Seychelles [43], stated the organising of after-school physical activity programmes by setting up health clubs, among other measures. Again, about a third [21,22,23,26,30,31,40,43,46,51,81,82,83] outlined the provision and access to adequate recreational facilities to promote active play. Algeria [21], Ethiopia [31], Mauritius [83], Seychelles [43], and South Africa [82] proposed strategies to promote and support active transport. Professional development and or trained instructors for school staff, including physical education teachers and school doctors was the focus of Algeria [21], Liberia [78], and Mauritius [83].

#### 3.2.1. Family

The key policy actions at the family level were the provision of educational materials and programmes, sensitisation to promote healthy lifestyles, promotion of breastfeeding, and home vegetable gardens. Nearly one-half of the countries, including Liberia, Mauritius, Morocco, Namibia, Seychelles, and South Africa, addressed the promotion of adequate infant and young child feeding, including exclusive breastfeeding with continued breastfeeding and adequate complementary feeding. Lesotho [36], Mauritius [70] and South Africa [82] addressed the promotion of home vegetable gardens, while Algeria [21], Burkina Faso [76], Ghana [33], Guinea-Bissau [74], Liberia [78], and Tunisia [81] targeted the sensitisation of parents to healthy diets and the importance of physical activity for health.

#### 3.2.2. Community

The majority targeted health promotion and awareness campaigns for healthy lifestyles. Botswana [22], Ghana [33] and Tunisia [81] proposed financial incentives for healthy lifestyles, such as rewards for active transport. Ghana, Mauritius, South Africa, Tunisia proposed the role models for physical activity. Ten countries proposed policy measures on the formation of private and public physical activity clubs, organisation of games and sports, and development of physical activity programmes. Thirteen countries proposed the production of and access to healthy foods, such as fruits and vegetables, through community initiatives.

### 3.3. Key Policy Interventions at the Macro-Scale

The policy measures at the macro-level included the production, provision, and access to healthy foods, such as fruits and vegetables; health promotion; regulation of processed foods; regulation of marketing of unhealthy foods and beverages; fiscal policies; development, implementation and strengthening of transport policies to improve the built and natural environments; and national physical activity guidelines. By targeting the food industry, 14 countries, including Benin, Botswana, Cote d’Ivoire, Egypt, Kenya, Morocco, Nigeria, Seychelles, and South Africa, outlined policy actions on the regulation of sugars, fats, and salt in processed foods. Furthermore, regulation of the marketing of unhealthy foods and beverages, especially to children, was addressed in 11 countries [21,22,30,33,35,40,43,49,70,74,82].

Thirteen countries [22,26,30,31,33,34,35,36,43,49,59,82,84] stated fiscal policies to promote healthier diets and discourage unhealthy diets. For instance, the sugar tax was one such strategy by South Africa [82] and Mauritius [59], while Liberia [84] and Egypt [30] mentioned the taxation of sugar-sweetened beverages in general. Other countries stated that subsidies would be introduced to promote the production and accessibility of fruits and vegetables. For example, Lesotho [36] specified the exemption of fruits and vegetables from taxation, while Guinea [34] proposed subsidies on local production of fruits and vegetables. Additionally, 13 countries [21,30,31,33,35,40,43,48,49,70,72,81,82] highlighted food and nutrition labelling through point of sale, product package, and front of pack labelling to provide adequate nutrition information to the consumer.

Several strategies were outlined to promote physical activity. The strengthening and implementation of transport policies to improve the built and natural environments were the main strategies outlined in 31 countries. The provision of and access to adequate recreational facilities to promote active play was addressed in 15 countries, including Algeria [21], Benin [23], Cote d’Ivoire [29], Ghana [33], Madagascar [37], South Africa [82] Togo [47], and Zambia [48]. Furthermore, the availability of safe walking paths, cycling lanes, and public transport were outlined in 16 countries, including Angola, Botswana, Congo, Egypt, Madagascar, Morocco, Nigeria, Seychelles, and Togo. In addition, other countries, including Cote d’Ivoire, Egypt, Ethiopia, Mauritius, Seychelles, and South Africa, outlined the development, strengthening or implementation of national physical activity guidelines and plans. Financial incentives for healthy lifestyles, such as subsidies on sports equipment, was the focus of Botswana [22], Ghana [33] and Tunisia [81].

### 3.4. Policy Domains

Majority of the policy interventions were legislative, sociocultural, and physical domains at both scales, with only two under the economic domain at the macro level.

## 4. Discussion

This scoping study was conducted to assess the nature and extent of policies related to overweight/obesity prevention in Africa and to assess how they align with the international guidelines. Despite the absence of standalone policies on obesity prevention—only two—the results highlight the availability of national policies targeted at promoting healthy diets and physical activity on the micro and macro scales. The evidence-based recommended population-based strategies for obesity and NCDs prevention [15,16,17] include interventions to reduce the main shared modifiable risk factors for NCDs. For example, objective 3 of the WHO Action Plan for the Global Strategy for the Prevention and Control of Non-communicable Diseases 2008–2013) proposed actions for Member States include: “promote interventions to reduce the main shared modifiable risk factors including unhealthy diets and physical inactivity”. The strategies involve the provision and promotion of healthy choices, which in the context of this review include policy actions to increase the intake of fruits and vegetables, decrease the intake of sugar-sweetened beverages, and promote physical activity for all through multi-sectoral actions that target multiple settings.

In the current review, the policy measures are generally in alignment with global strategies and recommendations. These documents underscore the importance of multi-sectoral collaborations in creating supportive environments. Many countries have proposed policy initiatives to address unhealthy diets and physical inactivity. The findings from the present review demonstrate a significant improvement relative to the 2013 findings by Lachat and colleagues [85], who observed a low coverage of diet and physical activity policies in African countries. Moreover, there are overlaps and interactions in the application of the framework. Except for the economic domain, the physical, legislative, and sociocultural domains were largely featured.

At the macro level, diet-related economic or fiscal policy initiatives by many governments have had strong opposition, mainly by the food and beverage industry [86,87]. Nonetheless, the evidence from high and low-to-middle income countries demonstrates the feasibility and effectiveness of these initiatives in providing supportive environments. There is considerable evidence that food taxes and subsidies encourage healthy food choices, food preferences and consumption, and subsequent reduction in BMI, obesity, and chronic diseases [13,86,88,89].

In their systematic review of high-income countries, Thow and colleagues [89] found that subsidies on healthy foods increased the purchase and consumption of these foods, particularly fruits and vegetables, by a lower margin relative to the subsidy; however, the overall effect on energy intake was inconsistent [89]. In the same analysis, the taxation of sugar-sweetened beverages reduced the consumption as well as the energy intake of these beverages proportional to the tax component [89]. In another meta-analysis involving France, Brazil, Mexico and the U.S.A. [88], the taxation of sugar-sweetened beverages increased the demand for healthier alternatives, such as milk, and also led to a reduction in the prevalence of overweight and obesity [88]. Limited evidence from Africa indicates that the implementation of the sugar tax, the Health Promotion Levy in 2018 in South Africa, has impacted product reformation, price increase, and reduction in the purchases of sugar-sweetened beverages, especially by low socioeconomic households [90,91].

Nutrition labelling is key to product reformation and provides adequate information to consumers at the point of sale, thereby encouraging healthy food choices. Nutrition labelling comes in variant forms, including front-of-package labelling and the traffic light system. The traffic light system, where the contents of sugar, salt, and total fats of packaged foods are indicated using colours, is recommended as one of the effective strategies in the prevention of obesity. Nutrition labelling may result in increased purchases of healthy food options, which may influence dietary quality [92,93].

Food advertising and marketing, especially to children, may influence their preferences and dietary intake and subsequently their weight status [94,95]. For example, evidence from a systematic review and meta-analysis [95] suggests that acute advertising exposure to unhealthy foods is significantly associated with greater food intake in children, but not in adults.

At the micro-level, food-based guidelines are relevant to ensure appropriate food standards. African countries have explicitly stated policy measures to ensure appropriate food standards in settings such as schools, restaurants, and fast food outlets. Comprehensive school-based health and nutrition policies are related to healthier eating habits, improved physical activity, and decreased rates of obesity. For example, limiting children’s access to foods that are high in fat and sugars and increasing access to healthy alternatives at the school are associated with improved dietary intake [96,97]. Furthermore, menu labelling at restaurants and fast food outlets informs consumers of the energy contents of menu items to make an informed decision at the point of purchase and is related to the purchase of low-calorie food [98].

The main policy interventions to promote physical activity are the provision of and access to adequate recreational facilities to promote active play, and the availability of safe walking paths, cycling lanes, and public transport, among others. Policy interventions at the macro level target the built and natural environments, public transport, access to parks and recreational facilities, and mass media campaigns for attitude and behaviour changes. Mass media campaigns and large-scale public participation events, for example, can be effective when linked to specific programmes, such as urban design and infrastructure, and land use to promote walking and cycling for exercise [99].

At the micro scale, these include recreational facilities at schools, and design features in institutions, communities, and buildings, such as stairways. Point-of-decision prompts, such as health and motivational messages at the base of stairways or the elevator, have shown moderate results, as they serve as reminders [99]. Unless the population is educated about the importance of active living, the availability of these alone might not produce the expected impact.

Addressing the global obesity pandemic through effective implementation of the proposed national policies and programmes is critical in meeting the United Nations Sustainable Development Goals (SDGs) 3 of “good health and well-being to ensure healthy lives and promote well-being at all ages”. It is not adequate to have these policies, as the presence of the documents is not indicative of implementation. The reported impact of obesity on global health, especially in light of the COVID-19 pandemic [100], calls for proactive and concerted actions by all relevant stakeholders, including governments, international partners, civil society, private sector involvement, and non-governmental organisations.

There are strengths of the present study. This is the only study on national policies in African countries that provides information on the available national documents outlining key policy interventions to address unhealthy diets, physical inactivity, and obesity. Other policy analyses have focused on a few selected African countries [101] or implementations of NCD policies [102]. Additionally, an attempt was made to include nearly all African countries, regardless of language. A source of limitation was the limited number of standalone policies on obesity prevention. Another potential limitation could be the categorisation of the key policy interventions at the domains and scales, as some of the initiatives tended to overlap and interact within the framework. Moreover, due to the anticipated volume of available documents, the searches were limited to national documents. Finally, language was another challenge that was addressed by resorting to online language translation services.

## 5. Conclusions

This study has provided information on the extent and nature of policies and programmes related to overweight and obesity prevention in Africa; it can be useful as a first point of call for policymakers on unhealthy diets, physical inactivity, and obesity policy documents. We found that the policy initiatives broadly align with global strategies and recommendations. The overlapping and interactions in the initiatives demonstrate the importance of multi-sectoral partnerships in providing supportive environments for healthy behaviours. It is recommended that a comprehensive systematic review and impact study of available national policies be conducted; this should include implementation, monitoring and evaluation, where applicable. The results should be made available in a regional repository.

## Figures and Tables

**Table 1:** Analysis grid for environments linked to obesity (ANGELO).

	Macro-Environment, Diet-Related and Physical Activity-Related (National, Regional, Sectors, Food Industries, Media, etc.)	Micro-Environment, Diet-Related and Physical Activity (Homes, Schools, Community Groups, Food Retailers, etc.)
Physical	What is available? Example: facilities, built environment, training opportunities, nutrition and exercise expertise, information.	
Economic	What are the monetary cost/factor influences and consequences? Example: taxes and subsidies.	
Legislative	What are the statutory provisions, rules and legal guidance, policy messages?	
Socio-cultural	What are the attitudes, beliefs, perceptions, and values?	

Adapted from Swinburn et al. (1999) [20].

**Table 2:** Categorisation of key policy interventions related to unhealthy diets, physical inactivity, and overweight/obesity prevention.

	Policy intervention	Environment	Country
Micro-scale			
School			
Nutrition and diet-related	Provision of healthy school meals (provision of, and access to like fruits and vegetables)	Physical/ Legislative	Algeria, Benin, Botswana, Chad, Egypt, Gabon, Ghana, Guinea-Bissau, Kenya, Liberia, Mauritius, Morocco, Mozambique, Seychelles, South Africa, Tanzania, Tunisia, Zambia
	Promotion of school vegetable gardens	Legislative	Algeria, Burkina Faso, Gabon, Guinea-Bissau, Rwanda, Tunisia.
	Restricting marketing of unhealthy foods and beverages	Legislative	Algeria, Ghana, Mauritius, South Africa, Tunisia
	Food supply near schools (limits on refined sugars, fats, and salt	Legislative	Algeria, South Africa
	Strengthen nutrition education	Legislative/Sociocultural	Algeria, Angola, Botswana, Burkina Faso, Cameroon, Egypt, Gabon, Gambia, Ghana, Guinea-Bissau, Guinea, Kenya, Liberia, Madagascar, Mauritius, Morocco, Rwanda, Seychelles, Sierra Leone, South Africa, Tanzania, Tunisia
	Professional development for teachers and school canteen staff, etc.	Physical/ Sociocultural	Algeria, Burkina Faso, Guinea-Bissau, Tanzania
	Monitoring of BMI	Legislative	Algeria, Botswana, Zambia
Physical activity	Mandatory/strengthen physical education and activity in the curriculum	Legislative/Sociocultural	Algeria, Angola, Botswana, Chad, Egypt, Ghana, Guinea-Bissau, Kenya, Lesotho, Liberia, Madagascar, Mauritius, Morocco, Sierra Leone, Seychelles, South Africa, Togo, Tunisia, Zambia
	After-school physical activity programmes	Physical/ Legislative	Mauritius, Seychelles
	Provision of, and access to adequate recreational facilities	Physical	Angola, Algeria, Benin, Botswana, Chad, Egypt, Ethiopia, Mauritius, Nigeria, Seychelles, South Africa, Tanzania, Tunisia
	Promote and support active transport	Physical/ Legislative	Algeria, Ethiopia, Mauritius, Seychelles, South Africa
	Professional development/trained instructors for school staff including PE teachers and school doctors	Physical/ Sociocultural	Algeria, Liberia, Mauritius
Family	Educational materials/programmes/sensitisation to promote healthy lifestyles	Physical/ Sociocultural	Algeria, Burkina Faso, Ghana, Guinea-Bissau, Liberia, Tunisia
	Promotion of breastfeeding	Physical/ Legislative/Sociocultural	Algeria, Botswana, Cameroon, Chad, Egypt, Gabon, Gambia, Guinea-Bissau, Kenya, Liberia, Malawi, Mauritania, Mauritius, Morocco, Namibia, Nigeria, Rwanda, Seychelles, South Africa, Togo, Zambia, Zimbabwe
	Promotion of vegetable gardens	Physical/ Sociocultural	Lesotho, Mauritius, South Africa
Community	Provision of, and access to adequate recreational facilities	Physical/Legislative	Algeria, Benin, Cameroon, Cote d’Ivoire, Gambia, Ghana, Guinea, Madagascar, Mauritius, Nigeria, Sierra Leone, South Africa, Togo, Tunisia, Zambia
	Provision of, and access to safe walking paths, cycling lanes, public transport, etc.	Physical/ Legislative	Angola, Algeria, Botswana, Congo, Chad, Egypt, Ghana, Madagascar, Mauritius, Morocco, Nigeria, Seychelles, Sierra Leone, South Africa, Togo, Zambia
	Physical activity clubs/organisation of games and sports	Sociocultural	Algeria, Botswana, Egypt, Chad, Ghana, Guinea-Bissau, Mauritius, South Africa, Togo, Tunisia
	Health promotion/awareness campaigns of healthy lifestyles (healthy foods/physical activity)	Legislative/ Sociocultural	Angola, Algeria, Benin, Burkina Faso, Cote d’Ivoire, Chad, Central African Republic, Egypt, Ethiopia, Gabon, Gambia, Ghana, Guinea-Bissau, Guinea, Kenya, Lesotho, Liberia, Madagascar, Malawi, Mauritania, Mauritius, Morocco, Namibia, Niger, Nigeria, Seychelles, Sierra Leone, South Africa, Swaziland, Tanzania, Togo, Zambia, Zimbabwe
	Financial incentives for healthy lifestyle (rewards for active transport)	Economic	Botswana, Ghana, Tunisia,
	Production, provision of, and access to healthy foods like fruits and vegetables	Physical/ Sociocultural/ Legislative	Benin, Chad, Congo, Cote d’Ivoire, Ethiopia, Guinea-Bissau, Guinea, Liberia, Madagascar, Mauritius, Seychelles, South Africa, Togo
	Role models for physical activity	Sociocultural	Ghana, Mauritius, South Africa, Tunisia
Macro-scale			
	Health promotion/awareness campaigns of healthy lifestyles (healthy foods/physical activity)	Legislative/ Sociocultural	Angola, Algeria, Benin, Burkina Faso, Cote d’Ivoire, Chad, Central African Republic, Egypt, Ethiopia, Gabon, Gambia, Ghana, Guinea-Bissau, Guinea, Kenya, Lesotho, Liberia, Madagascar, Malawi, Mauritania, Mauritius, Morocco, Namibia, Niger, Nigeria, Seychelles, Sierra Leone, South Africa, Swaziland, Tanzania, Togo, Zambia, Zimbabwe
	Educational material/training relevant stakeholders for healthy lifestyles e.g., consumers, food manufacturers, NGO, etc.	Physical/ Sociocultural	Algeria, Cameroon, Chad, Egypt, Gabon, Ghana, Mauritius, Morocco, Seychelles, South Africa, Tunisia
	Financial incentives for healthy lifestyle (e.g., subsidies on sports equipment and bicycles	Economic	Botswana, Ghana, Tunisia
	National physical activity guidelines/plans	Legislative	Cote d’Ivoire, Egypt, Ethiopia, Ghana, Mauritius, Nigeria, Seychelles, South Africa, Zambia
	Provision of, and access to adequate recreational facilities	Physical/Legislative	Benin, Chad, Congo, Cote d’Ivoire, Ethiopia, Guinea-Bissau, Guinea, Liberia, Madagascar, Mauritius, Seychelles, South Africa, Togo
	Food taxes and subsidies to promote healthier diets	Economic	Botswana, Chad, Egypt, Ethiopia, Ghana, Guinea, Kenya, Lesotho, Liberia, Mauritius, Morocco, Seychelles, South Africa
	Marketing of unhealthy foods and beverages especially to children	Legislative	Algeria, Botswana, Egypt, Ghana, Guinea-Bissau, Kenya, Mauritius, Morocco, Nigeria, Seychelles, South Africa
	Food and nutrition labelling	Sociocultural/ Legislative	Algeria, Egypt, Ethiopia, Ghana, Kenya, Mauritius, Morocco, Namibia, Nigeria, Seychelles, South Africa, Tunisia, Zambia
	Production, provision of, and access to healthy foods like fruits and vegetables	Physical/ Sociocultural/ Legislative	Benin, Chad, Congo, Cote d’Ivoire, Ethiopia, Guinea-Bissau, Guinea, Liberia, Madagascar, Mauritius, Seychelles, South Africa, Togo
	Regulation of sugars, fats, and salt in processed foods	Legislative/ Sociocultural	Benin, Botswana, Congo, Cote d’Ivoire, Chad, Egypt, Ghana, Kenya, Madagascar, Morocco, Nigeria, Seychelles, South Africa, Zambia

## Supplementary Materials

The following are available online at https://www.mdpi.com/article/10.3390/nu13114028/s1, Table S1: National policy, actions, programmes, and strategies on diet and physical activity to prevent obesity/non-communicable diseases in African countries.
