# Supplementary material for: A Scoping Review of Policies Related to the Prevention and Control of Overweight and Obesity in Africa
Source: Nutrients. 2021 Nov 11;13(11):4028. doi: 10.3390/nu13114028 (PMC8625107; doi:10.3390/nu13114028)
Supplement: Supplementary file 1 [file nutrients-13-04028-s001.zip › nutrients-1350947-supplementary.pdf]

Supplementary Table 1: National policies, actions, programmes, and strategies on diet and physical activity to prevent obesity/non-communicable diseases in African countries

Country	Title and type of document	Year
Algeria	Multi-sectoral Integrated Strategic Plan for Control of Risk Factors of Non-Communicable Diseases	2015-2019
Angola	Health Development Plan	2012-2025
Benin	Integrated Strategic Plan for the Control of Non-communicable Diseases	2014-2018
Botswana	Multi-sectoral Strategy for the Prevention and Control of Non-Communicable Diseases	2017-2022
Burkina Faso	Integrated Strategic Plan for Control of Non-communicable Diseases<br>Nutrition Policy	2016-2020*<br>2016
Burundi	Multi-sectoral Strategic Plan for Food Safety and Nutrition<br>Health Development Plan	2014-2017*<br>2011-2015*
Cameroon	Health Development Plan<br>Food and Nutrition Policy	2016-2020<br>2007-2011
Cape Verde	Health Development Plan	2012-2016*
Central African Republic	Policy for the Prevention and Control of Non-communicable Diseases	2014
Chad	Multi-sectoral Plan to fight and control non-communicable Diseases	2017-2021
Comoros	Strategic Document for Prevention and Control of Non-communicable Diseases	2013*
Congo	Integrated Plan for Control of Non-communicable Diseases	2013-2017
Cote d'Ivoire (Ivory Coast)	Integrated Strategic Plan for Prevention and Management of Non-communicable Diseases	2015-2019
DR of the Congo	Health Development Plan	2014*
Djibouti	Health Development Plan	2013-2017*
Egypt	Multi-sectoral Action Plan For Non-communicable Diseases Prevention and Control	2018-2022
Ethiopia	Strategic Action Plan for Prevention and Control of Non-communicable Diseases	2014-2016
Eritrea	Non-communicable Diseases Policy	2008*
Gabon	Policy on Food Security and Nutrition	2017-2025
Gambia	Health Sector Strategic Plan<br>Nutrition Policy	2014-2020*<br>2010-2020
Ghana	Strategy for the Management, Prevention and Control of Chronic Non-Communicable Diseases in Ghana	2012-2016
Guinea-Bissau	National Nutrition Policy	2008-2017
Guinea	Integrated Programme for the Prevention and Control of Non-communicable Diseases	2011-2015
Kenya	Strategy for the Prevention and Control of Non Communicable Diseases	2015-2020
Lesotho	Multi-sectoral Integrated Strategic Plan for the Prevention and Control of Non-communicable Diseases	2014-2020
Liberia	Policy and Strategic Plan on Health Promotion<br>Food Security and Nutrition Strategy	2016-2021<br>2008
Madagascar	National Policy for Prevention and fight against Integrated non-communicable chronic Diseases<br>Action Plan for Nutrition	 2017-2021*
Malawi	Health Sector Strategic Plan II<br>Multi-sector Nutrition Policy	2017-2022*<br>2018-2022
Mali	Policy to fight against non-Communicable Diseases	2013*
Mauritania	Health Development Plan	2012-2020
Mauritius	Health Sector Strategy<br>Plan of Action for Nutrition Final<br>Action Plan on Physical Activity	2017-2021<br>2009-2010<br>2011-2014

Morocco	Multi-sectoral Integrated Strategic Plan for the Prevention and Control of Non-communicable Diseases	2016-2025
Mozambique	Strategic Plan for Prevention and Control of Non-communicable Diseases	2008-2014
Namibia	Health Policy Framework<br>Strategic Plan for Nutrition	2010-2020*<br>2011-2015
Niger	Integrated Strategic Plan for Prevention and Control of Chronic Non-communicable Diseases	2012
Nigeria	Policy and Strategic Plan of Action On Non-communicable Diseases	2013
Rwanda	Non-communicable Diseases Policy<br>Food and Nutrition Policy	2015<br>2013-2018
Sao Tome and Principe	Health Development Plan	2017-2021
Senegal	Health Development Plan	2009-2018*
Seychelles	Strategy for the Prevention and Control of Non-Communicable Diseases	2016-2025
Sierra Leone	Non-communicable Diseases Strategic Plan<br>Food and Nutrition Security Policy	2013-2017<br>2012–2016*
Somaliland	National Health Policy<br>Health Sector Strategic Plan	2011*<br>2013-2016
South Africa	Strategy for the Prevention and Control of Obesity	2015-2020
South Sudan	Health Policy Framework and Work Plan	2013-2016*
Sudan	25 years Strategic Plan for Health Sector	2003-2027*
Swaziland	Non-communicable Diseases Prevention and Control Policy	2016
Tanzania	Strategic and Action Plan for the Prevention and Control of Non-communicable Diseases	2016-2020
Togo	Integrated Policy and Strategic Plan to fight against non-communicable diseases	2012-2015
Tunisia	Strategy for Prevention and Control of Obesity	2013-2017
Uganda	Health Sector Development Plan<br><br>Nutrition Action Plan	2015/16 *<br>2019/20<br>2011-2016*
Zambia	Strategic Plan Non-communicable Diseases and their risk factors	2013-2016
Zimbabwe	National Health Strategy	2016-2020

DR: Democratic Republic; * denotes documents that were excluded from the review
